# Announcement: Standards and Ontologies for Functional Genomics (SOFG) Conference

**DOI:** 10.1002/cfg.188

**Published:** 2002-08


					Announcement
Standards and Ontologies for Functional Genomics
(SOFG) Conference
- towards unified ontologies for biology and biomedicine
17-20 November 2002
Hinxton Hall Conference Centre, The Wellcome Trust Genome
Campus, Hinxton, Cambridge, UK
The goal of this conference is to bring together scientists who are developing
standards and ontologies for describing microarray and other high throughput
functional genomics experiments. The topics will include:
* The Gene Ontology (GO) project
* Anatomy and phenotype ontologies
* Disease ontologies
* Chemical compounds and treatments
* Inter-ontology mapping
* Tools for ontology development
Confirmed Keynote Speakers
Ken Buetow, NCI, USA
Winston Hide, SANBI, South Africa
Peter Karp, SRI International, USA
Lincoln Stein, CSHL, USA
For more information visit:
www.wellcome.ac.uk/hinxton/sofg
Programme Committee
Michael Ashburner, University of Cambridge, UK (Chair)
Cathy Ball, Stanford University, USA Victor Markowitz, Gene Logic, USA
Mike Bittner, NHGRI, USA John Quackenbush, TIGR, USA
Alvis Brazma, EMBL-EBI, UK Martin Ringwald, The Jackson Laboratories, USA
Duncan Davidson, MRC HGU, Edinburgh, UK Steffen Schulze-Kremer, RZPD, Germany
Robin McEntire, GSK, UK Paul Spellman, UC Berkeley, USA
Midori Harris, EMBL-EBI, UK Robert Stevens, University of Manchester, UK
Chris Stoeckert, University of Pennsylvania, USA
The conference is supported by the Wellcome Trust and the Microarray and Gene Expression
Database group (MGED, www.mged.org).

				

